# Former orphan riboswitches reveal unexplored areas of bacterial metabolism, signaling, and gene control processes

**DOI:** 10.1261/rna.074997.120

**Published:** 2020-06

**Authors:** Madeline E. Sherlock, Ronald R. Breaker

**Affiliations:** 1 Department of Molecular Biophysics and Biochemistry, Yale University, New Haven, Connecticut 06520, USA; 2 Department of Molecular, Cellular and Developmental Biology, Yale University, New Haven, Connecticut 06520, USA; 3 Howard Hughes Medical Institute, Yale University, New Haven, Connecticut 06520, USA

**Keywords:** aptamer, *cis*-regulatory element, comparative sequence analysis, noncoding RNA, RNA motif

## Abstract

Comparative sequence analyses have been used to discover numerous classes of structured noncoding RNAs, some of which are riboswitches that specifically recognize small-molecule or elemental ion ligands and influence expression of adjacent downstream genes. Determining the correct identity of the ligand for a riboswitch candidate typically is aided by an understanding of the genes under its regulatory control. Riboswitches whose ligands were straightforward to identify have largely been associated with well-characterized metabolic pathways, such as coenzyme or amino acid biosynthesis. Riboswitch candidates whose ligands resist identification, collectively known as orphan riboswitches, are often associated with genes coding for proteins of unknown function, or genes for various proteins with no established link to one another. The cognate ligands for 16 former orphan riboswitch motifs have been identified to date. The successful pursuit of the ligands for these classes has provided insight into areas of biology that are not yet fully explored, such as ion homeostasis, signaling networks, and other previously underappreciated biochemical or physiological processes. Herein we discuss the strategies and methods used to match ligands with orphan riboswitch classes, and overview the lessons learned to inform and motivate ongoing efforts to identify ligands for the many remaining candidates.

## INTRODUCTION

**FIGURE 1.**
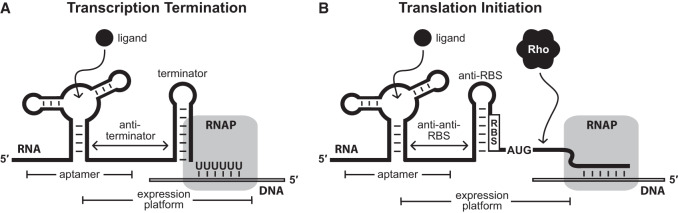
Riboswitch-regulated gene expression is most commonly mediated through transcription termination and translation initiation control mechanisms. (*A*) A model for a transcriptional “OFF” riboswitch, wherein ligand binding promotes formation of an intrinsic terminator stem to cease transcription elongation. An alternative “anti-terminator” stem forms in the absence of ligand binding that precludes terminator stem formation. (*B*) A model for a translational “OFF” riboswitch, wherein ligand binding sequesters the ribosome binding site (RBS) to prevent translation initiation. Presumably, the portion of the mRNA that lacks the protection of ribosomes permits Rho termination binding and subsequent transcription termination. An alternative “anti-anti-RBS” stem forms in the absence of ligand binding that prevents anti-RBS stem formation. Figure adapted from a previous publication (Breaker 2012).

Riboswitches reside in the noncoding portion of mRNAs and bind their corresponding ligands to control gene expression (Roth and Breaker 2009; Breaker 2011; Serganov and Nudler 2013). These noncoding RNA domains are abundant in the eubacterial domain of life, where they usually reside in the 5′ untranslated region (UTR) of mRNAs directly preceding the open reading frame (ORF) whose expression they regulate (Roth and Breaker 2009; Sherwood and Henkin 2016). To accomplish their *cis*-regulatory role, each riboswitch sequence is almost always located on the same strand and oriented in the same direction as the coding region(s) under its control. They are unique compared to other regulatory RNAs, such as small RNAs (sRNAs), ribosomal protein leaders, and T box RNAs (Grundy and Henkin 2006; Waters and Storz 2009; Green et al. 2010; Gottesman and Storz 2011), in that riboswitches directly bind a small molecule or ion ligand to control gene expression. The relative abundance of the target ligand is responsible for determining whether the downstream genes are transcribed and/or translated (Fig. 1). Therefore, the identity of the cognate ligand for each riboswitch class is intrinsically linked to the functions of the genes it regulates (Winkler and Breaker 2005).

Ligands for known riboswitch classes include coenzymes, nucleotide derivatives, amino acids, sugars, and elemental ions (McCown et al. 2017). Riboswitches can function as “ON” or “OFF” switches in a manner dictated by the ligand they sense, and by the nature of the downstream gene or operon (Roth and Breaker 2009). For example, a high concentration of *S*-adenosylmethionine (SAM) is sensed by several riboswitch classes to repress SAM biosynthesis genes when this coenzyme is plentiful (Wang and Breaker 2008; Mirihana Arachchilage et al. 2018). Alternatively, high concentrations of Ni^2+^ or Co^2+^ trigger the NiCo riboswitch class to turn on genes encoding exporters for these ions, which are toxic at high concentrations (Furukawa et al. 2015). To regulate gene expression in a ligand-dependent manner, the riboswitch RNA must be able to both specifically recognize its target ligand and communicate this binding event to the cellular machinery responsible for gene expression.

Most riboswitches use two overlapping domains to accomplish its tasks: a selective ligand-binding aptamer region and an expression platform that commonly influences the function of the cell's transcription, translation, or RNA processing machinery (Fig. 1; Breaker 2012). Aptamer domains usually appear first in the RNA transcript (5′-proximal), and carry conserved nucleotides and structural features that are characteristic of each riboswitch class (Barrick and Breaker 2007; McCown et al. 2017). Typically, the most highly conserved nucleotides reside in and around the ligand binding pocket, and play roles ranging from hydrogen bonding with the ligand to coordinating metal ions for charge neutralization (Barrick and Breaker 2007; Serganov and Patel 2012). In some cases, as noted above for SAM riboswitches, several entirely distinct aptamer architectures are used to sense the same ligand, and each of these architectures is considered a different class. In other cases, a slight alteration in nucleotide sequence to an aptamer class yields a similar architecture, but the resulting aptamer has switched its ligand specificity and regulates genes for a different biological process. These also are considered separate riboswitch classes due to the change in ligand specificity.

In contrast to the aptamer domain, the sequence of the trailing expression platform is usually poorly conserved. The expression platform dictates gene expression based on aptamer occupancy (Roth and Breaker 2009), usually by forming alternative base-pairing interactions in a ligand-dependent fashion. Thus, almost without exception, expression platforms exhibit considerable sequence diversity because the base-pair identities can drift over evolutionary time. One prominent exception is the riboswitch class for hydroxymethyl-pyrimidine pyrophosphate (HMP-PP), a thiamin pyrophosphate (TPP) biosynthetic intermediate (Atilho et al. 2019; Stav et al. 2019). As discussed in more detail later, the aptamer of this unusual riboswitch class almost entirely overlaps the expression platform, which requires most of the riboswitch to remain highly conserved.

Riboswitch regulation typically occurs either during transcription by influencing the stability of an intrinsic terminator stem, or during translation initiation by exposing or sequestering the ribosome binding site and/or start codon (Fig. 1; Barrick and Breaker 2007; Roth and Breaker 2009; Sherwood and Henkin 2016; Breaker 2018). The expression platforms for these common mechanisms are relatively easy to recognize by using standard sequence and structural analysis methods. However, other mechanisms including ribozyme and RNA processing regulation are also used (Breaker 2012), which can be more difficult to recognize or predict.

**FIGURE 2.**
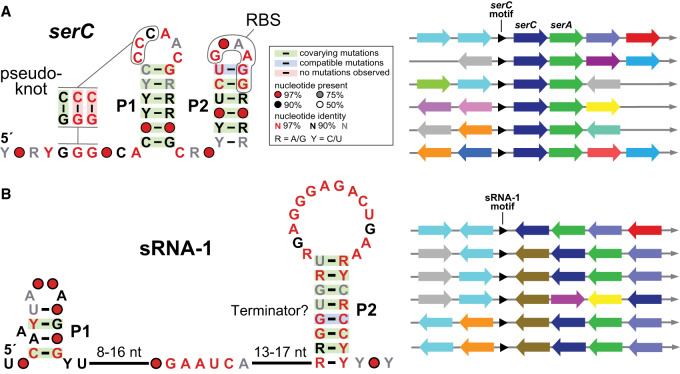
Identifying strong riboswitch candidates based on characteristics of structured nucleic acid motifs. (*A*) Consensus model and representative genomic contexts of the *serC* motif, a long-standing orphan riboswitch candidate (Corbino et al. 2005; Greenlee et al. 2018). (*Left*) RNA motifs with a high probability of functioning as riboswitches, such as *serC*, have conserved nucleotides located within a complex secondary structure supported by covariation of base-pairing. In addition, riboswitches frequently carry sequences or structures consistent with expression platform function, such as a ribosome binding site (RBS) located within or near to the aptamer. (*Right*) Representative genetic contexts for the *serC* motif. Strong riboswitch candidates are consistently located upstream and oriented in the same direction as the ORFs whose expression they regulate. The genes under riboswitch regulation typically have a metabolic theme, such as serine metabolism in this example (SerC, phosphoserine aminotransferase; SerA, 3-phosphoglycerate dehydrogenase). (*B*) Consensus model and representative genomic contexts of the sRNA-1 motif. (*Left*) RNA motifs with a low probability of functioning as metabolite-binding riboswitches, such as sRNA-1, commonly have simpler or weakly supported secondary structure models with few conserved nucleotides. The sRNA-1 motif does carry a large number of conserved nucleotides, and the predominant structural feature, the P2 stem, could be an intrinsic terminator (strong stem followed by a run of U nucleotides) that functions as an expression platform. (*Right*) Although sRNA-1 exhibits some promising sequence and structural features, its genomic context is not consistent with a *cis*-regulatory element. Therefore, this motif appears to be a stronger candidate for function as a bacterial small RNA regulator (Stav et al. 2019).

Characteristics of a strong riboswitch candidate can be readily observed by examining representatives from a variety of species. Comparative sequence analysis algorithms (e.g., Corbino et al. 2005; Fuchs et al. 2006; Weinberg et al. 2007, 2010, 2017a; Meyer et al. 2009; Ames et al. 2010) can be used to identify conserved nucleotide sequences and secondary structure features among the representatives of a specific class. This is achieved by first aligning the sequences of the representatives to maximize nucleotide sequence identity. In addition, any differences in the sequences can be examined for nucleotide covariation, which serves as evidence for the presence of important base-paired substructures (Fig. 2A). The presence of conserved nucleotides in the loops, linkers, and bulges that connect the base-paired substructures are evidence for complex tertiary structure formation. Often, more complex structural features such as multistem junctions and pseudoknots are present. However, these features are also consistent with some other types of noncoding RNAs (ncRNAs), including ribozymes and sRNAs (Fig. 2B).

The key characteristic distinguishing riboswitches from most other ncRNAs is genetic context. Riboswitches are *cis*-regulatory and, therefore, consistently appear in the proper orientation upstream of the same gene type or genes involved in the same biological process in multiple species. However, not all *cis*-regulatory RNA structures are riboswitches. These other types usually rely on interactions with a protein or RNA factor to regulate gene expression, and often do not display the structural complexity that is typical of RNA receptors for small-molecule or ion ligands (Fig. 2).

Given the subtleties of the characteristics of various ncRNA classes, and occasionally structured DNA classes (e.g., Roth and Breaker 2013; Cury et al. 2016), it is not always straightforward to distinguish a promising riboswitch candidate from other types of structured nucleic acid motifs. Even when taking care to focus validation experiments on the strongest candidates, other factors can intervene to complicate matchmaking between a riboswitch and its ligand. Herein, we review the outcomes of experimental efforts that have established the ligands for orphan riboswitch candidates. Also, given that many additional classes of orphan riboswitches remain to be matched with their natural ligands, we particularly note the experimental challenges encountered in these validation efforts. The strategies used to overcome these roadblocks are noted with the expectation that they may be applicable to future orphan riboswitch validation studies.

## RIBOSWITCH DISCOVERY

The first natural riboswitches to be experimentally validated were pursued based on some unexplained genetic and bioinformatic data. We (Nahvi et al. 2002; Winkler et al. 2002a,b) and others (Mironov et al. 2002) initially focused on analyzing the regulation of genes for which metabolite-dependent regulation involving the 5′ leader sequence had been proposed, but evidence for the involvement of a protein factor was suspiciously absent. Evidence that we now understand to be consistent with riboswitch function was published in some instances many years before a riboswitch class was convincingly validated. Such early findings were relevant for riboswitches that respond to coenzyme B_12_ (AdoCbl) (Nou and Kadner 2000), TPP (Miranda-Rios et al. 2001), flavin mononucleotide (FMN) (Gelfand et al. 1999), guanine (Ebbole and Zalkin 1987), SAM (Grundy and Henkin 1998), lysine (Hirshfield and Zamecnik 1972; Kochhar and Paulus 1996), and molybdenum cofactor (MoCo) (Anderson et al. 2000). These initial publications hinted at the existence of unusual regulatory systems and helped expose many of the initial riboswitch candidates.

In rapid succession, each of the storylines noted above were converted into proven riboswitch classes wherein the ligands and gene control mechanisms were reported. Relying on genetic experiments to identify riboswitch candidates had the immediate benefit of linking a riboswitch to its ligand. Unfortunately, simply searching the literature for possible new riboswitch candidates has long ago run its course. Also, there are severe limits to the utility of genetic searches for additional riboswitch classes. Most obvious is the fact that there are probably very few classes that remain undiscovered in well-studied and genetically tractable bacterial species.

To overcome these limitations, bioinformatic search strategies were developed to identify novel RNA motifs that might represent additional riboswitch classes (Barrick et al. 2004; Corbino et al. 2005; Fuchs et al. 2006; Weinberg et al. 2007, 2010, 2017a; Meyer et al. 2009; Ames et al. 2010). Such bioinformatic searches yield an alignment of individual sequence representatives of a particular class from different organisms, and reveal its most important sequence and structural features. Highly conserved nucleotide positions and base-pair interactions form a characteristic sequence and secondary structure consensus model, which can then be used to search genomic databases for additional representatives in diverse types of bacteria. By increasing the number of representatives for a given class, the consensus model can be refined and the data regarding genetic context can be increased, which together are useful for formulating hypotheses regarding the possible function of the motif (Fig. 2).

As comparative sequence analysis algorithms improved, this search strategy was used by several groups to identify entirely new riboswitch classes. For example, numerous representatives of eight novel RNA motifs were identified by comparing all intergenic regions from *Bacillus subtilis* with those from 90 additional bacterial species (Barrick et al. 2004). Seven of these eight motifs have since proven to represent distinct riboswitch classes (McCown et al. 2017; Nelson et al. 2017; Sherlock et al. 2018a,b, 2019). Since 2004, nearly all riboswitch class discoveries have relied on some form of comparative sequence analysis approach (e.g., Corbino et al. 2005; Fuchs et al. 2006; Weinberg et al. 2007, 2010, 2017a; Meyer et al. 2009; Ames et al. 2010). Some variant riboswitch classes have also been identified through bioinformatic analyses, wherein specific nucleotides near the binding pocket have been observed to mutate, thereby altering their ligand specificity compared to the original parent riboswitch class (Mandal and Breaker 2004; Kim et al. 2007; Nelson et al. 2017; Weinberg et al. 2017b).

For the first reported riboswitch classes (Mironov et al. 2002; Nahvi et al. 2002; Winkler et al. 2002a,b; Grundy et al. 2003; Sudarsan et al. 2003a), it was already known from published experiments that the associated genes were regulated by certain metabolites. An interesting outcome from the initial bioinformatics efforts to identify novel examples of riboswitch candidates was the discovery of multiple riboswitch candidates for which the identity of their cognate ligand was not immediately clear (Barrick et al. 2004). Furthermore, for some riboswitch candidates such as the *gcvT* RNA, the ligand was immediately predicted based on the involvement of the downstream genes in metabolic pathways related to glycine (Barrick et al. 2004; Mandal et al. 2004). However, the complex architecture of most members of this riboswitch class interfered with initial attempts to prove that glycine was being directly sensed (see additional discussion below). Similarly, efforts to confirm the identity of the cognate ligand for other motifs from this early collection of riboswitch candidates proved to be even more difficult (Block et al. 2010; Meyer et al. 2011), with some candidates resisting experimental validation for many years.

Protein receptors for which the ligand identity is undetermined are known as orphan receptors (Howard et al. 2001; Yoshida et al. 2012). Therefore, RNA motifs with a high likelihood to function as riboswitches but whose cognate ligands have not yet been identified were analogously termed orphan riboswitch candidates (Barrick et al. 2004; Dambach and Winkler 2009; Breaker 2011; Meyer et al. 2011; Greenlee et al. 2018). Although nearly all of the orphan riboswitch candidates from the initial large-scale bioinformatics discovery effort (Barrick et al. 2004) have now been experimentally validated, a considerable backlog of unresolved candidates remains to be explored. This list will undoubtedly grow as bioinformatics searches continue to reveal additional riboswitch candidates whose ligands are obscured to researchers for various reasons. In the sections below, we present details regarding successful orphan riboswitch studies and highlight some of the strategies that will likely be useful for those interested in resolving such mysteries in the future.

## ORPHAN RIBOSWITCHES

There are numerous reasons why a novel structured RNA motif might languish on the orphan riboswitch candidate list, but these usually become fully apparent only after the target has been identified. For example, information regarding the function of the protein products of riboswitch-regulated genes might be sparse, or the functions could be incorrectly assigned. Thus, gene annotations sometimes provide too few clues regarding the identity of the riboswitch ligand, or worse, they can be entirely misleading. Also, experimental problems, including the use of incomplete or misfolded RNA constructs, unstable or unavailable ligand candidates, or difficulties with genetic studies in certain bacteria have all plagued previous ligand validation studies.

**FIGURE 3.**
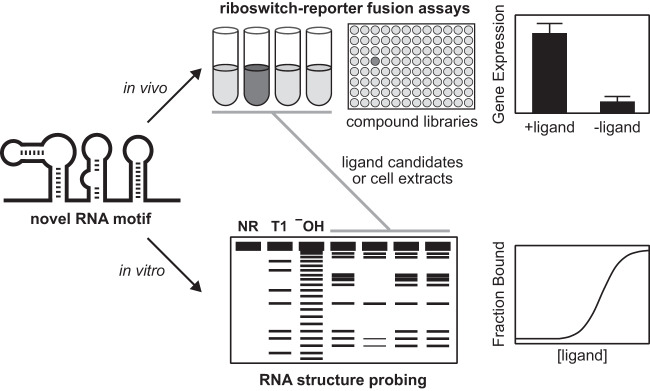
Schematic representation of major experimental strategies for riboswitch ligand discovery and validation. Candidate riboswitch aptamer motifs are typically discovered through comparative genomics analyses. Putative ligand compounds are evaluated with representatives of the riboswitch candidate using either an in vivo reporter gene fusion or an in vitro RNA structure probing assay. Other approaches, such as screening compound libraries and cellular extracts, have also been implemented when the riboswitch candidate fails to bind predicted ligand candidates. Once the ligand has been identified, additional experiments are performed to fully explore the riboswitch ligand interactions, such as measuring the affinity of the riboswitch RNA for its ligand and performing mutational analyses.

Occasionally, luck or serendipity is involved or even necessary in achieving an experimental breakthrough. Experimental methods can even be intentionally chosen to favor chance encounters with the natural ligand for a riboswitch candidate, and such combinatorial approaches have been proven useful for identifying the natural ligand for riboswitches (Fig. 3). These include the use of biochemical assays with many ligand candidates to evaluate RNA-ligand complex formation, such as screening cell extracts as a source of biologically relevant metabolites, or using riboswitch-reporter fusion constructs to examine various growth conditions. Because biochemical and genetic assay constructs could have flaws, several similar constructs, which either vary in length or are derived from different species are usually tested in parallel.

For the long-standing orphan riboswitch classes that have been validated to date, no single strategy worked (or could have worked) to discover the ligand for every candidate. In some cases, the functions of genes under the regulon of the orphan riboswitch class were reported subsequent to the discovery of the RNA motif, whereupon the ligand identity could finally be easily validated. For other classes, the ligands were fortuitously discovered through various experimental analyses, and key insights into previously unknown pathways and processes regulated by these riboswitch classes were gained only after the ligands were identified. Despite such difficulties, the biological insights gleaned through the validation of each of 16 former orphan riboswitch classes (Table 1) described in detail below, provides ample motivation to continue pursuing the functions of these other orphan riboswitch candidates.

**TABLE 1.**
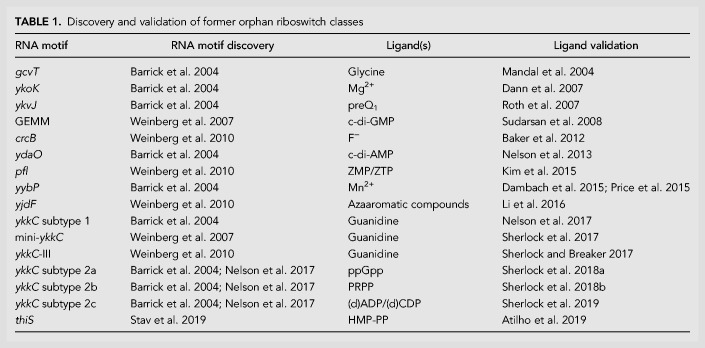
Discovery and validation of former orphan riboswitch classes

### *gcvT* motif RNAs: glycine riboswitches

The *gcvT* riboswitch candidate was published among the first wave of orphan riboswitch candidates discovered by using comparative sequence analysis (Barrick et al. 2004). It was readily apparent that *gcvT* motif RNAs had characteristics indicative of riboswitch function. Most informative was the fact that representatives were commonly found upstream of genes associated with glycine degradation, suggesting that this amino acid or another associated metabolites might serve as the natural ligand. Despite these strong clues, initial experimental validation efforts were confounded by the sophisticated structural and functional characteristics of most representatives. Specifically, experiments using a minimal *gcvT* motif construct failed to bind any ligand candidate tested.

A hint that *gcvT* motif RNAs were unusually complex was provided by the discovery of two highly similar versions of putative aptamer domains, called type I and type II (Barrick et al. 2004). Eventually, it was recognized that these two types frequently reside in tandem with each other and, when naturally present, this arrangement is necessary for their proper function (Mandal et al. 2004; Babina et al. 2017). By using extended RNA constructs that permit aptamer function, it was quickly determined that *gcvT* motif RNAs are highly selective aptamers for glycine (Barrick et al. 2004; Mandal et al. 2004), and that the two aptamers can work cooperatively to bind glycine and regulate gene expression in cells (Mandal et al. 2004; Kwon and Strobel 2008; Butler et al. 2011; Erion and Strobel 2011). Although the importance of cooperativity with this riboswitch class has since been questioned (Kladwang et al. 2012; Sherman et al. 2012), the recognition of cooperative function of certain constructs was a critical step along the path to experimental validation of glycine as the natural ligand.

The initial dissociation constant (*K*_D_) value measure for glycine binding in its tandem configuration was ∼10 μM (Mandal et al. 2004), but the disruption of one aptamer in a cooperative pair could degrade affinity. Presumably, this was the cause of the early binding assay failures when testing glycine. Also, some riboswitch aptamers that function independently exhibit relatively poor binding affinities because they are tuned to respond only to very high concentrations of ligands in cells. For example, riboswitches for glutamine have *K*_D_ values measured in the low mM range (Ames and Breaker 2011). Evidence for ligand binding will be missed by some assays if the concentrations tested fall substantially below these *K*_D_ values. Unfortunately, adding too much of a ligand candidate can cause experimental artifacts, and so researchers should take great care when designing such experiments and when passing judgment on the resulting data.

### *ykoK* motif RNAs: Mg^2+^-I riboswitches

Representatives of the *ykoK* riboswitch candidate (Barrick et al. 2004) were known to associate with cation transporters, especially those for Mg^2+^. The structure of this RNA motif is elaborate and often spans nearly 200 nt. In part, the unusual size and structural complexity of this candidate hindered its validation as a Mg^2+^ riboswitch class because it initially seemed unlikely for an RNA to require so many nucleotides to orchestrate a structural reconfiguration in response to changing Mg^2+^ concentrations. Essentially all RNAs are expected to at least subtly change their shapes when Mg^2+^ concentrations vary. This means that a Mg^2+^-dependent change in the shape of an RNA alone is insufficient to conclusively confirm the existence of a Mg^2+^-sensing riboswitch.

In vitro experiments typically used for riboswitch validation (Soukup and Breaker 1999; Wickiser et al. 2005; Regulski and Breaker 2008) use high levels of Mg^2+^ due to its well-established role in RNA structure stabilization (Misra and Draper 1998). With this added burden of proof, the validation of the *ykoK* motif as the Mg^2+^-I riboswitch class involved a combination of many techniques including in vitro transcription termination, multiple in vitro structure probing methods, in vivo riboswitch-*lacZ* reporter fusions, analytical ultracentrifugation, and a high-resolution crystal structure (Dann et al. 2007). These extensive studies revealed that this aptamer binds several Mg^2+^ ions in a highly cooperative fashion, which helps explain the necessity for such a complex aptamer structure. Notably, the validation of Mg^2+^ as the ligand for the *ykoK* orphan riboswitch class, and similarly the later discovery and validation of the Mg^2+^-II riboswitch class (Cromie et al. 2006; Groisman et al. 2009), provided the first evidence for an elemental ion to be the primary target for riboswitch RNAs. All previously validated ligands for riboswitches had been small-molecule metabolites.

### *ykvJ* motif RNAs: preQ_1_ riboswitches

Due to the relatively small size and simple structure of the *yvkJ* RNA motif, it was originally considered to be only a weak riboswitch candidate (Barrick et al. 2004). At the time, most known riboswitch aptamers consisted of multiple stems and were at least 100 nt in length. In comparison, *ykvJ* RNA motif representatives can be just 34 nt long and comprise a single stem loop followed by a tail of conserved nucleotides (Roth et al. 2007), some of which were later revealed to form a pseudoknot (Klein et al. 2009).

Despite its simple structure, the motif still possessed many qualities that implicated it as a *cis*-regulatory element. The genes most commonly associated with the motif include homologs of the *B. subtilis ykvJKLM* operon, which code for proteins whose functions had not yet been established. Initially, this prevented the formulation of well-founded hypotheses regarding possible ligands. Fortunately, the role of the *ykvJKLM* operon in the biosynthesis of queuosine from GTP was subsequently reported (Reader et al. 2004). It was then demonstrated that *ykvJ* RNAs specifically recognize prequeuosine-1 (preQ_1_), which is an intermediate in the queuosine biosynthesis pathway (Roth et al. 2007).

Because preQ_1_ had been established as the ligand for *ykvJ* RNAs, now termed the preQ_1_-I riboswitch class, it was also possible to validate the same molecule as the ligand for two additional candidate riboswitch classes discovered later. These distinct riboswitch classes, now termed preQ_1_-II and preQ_1_-III, are associated with the same biosynthetic operon but in different species (Meyer et al. 2008; McCown et al. 2014). The preQ_1_-I riboswitch class is an additional example of the frequent partnerships between riboswitches and ligands that are nucleotide-derived compounds (McCown et al. 2017). The discovery of preQ_1_-I riboswitches also provided evidence that exceedingly simple natural RNA structures can selectively and independently bind a small-molecule ligand to regulate gene expression.

### GEMM motif RNAs: c-di-GMP-I riboswitches

The GEMM (genes for the environment, for membranes, and for motility) RNA motif (Weinberg et al. 2007) was the first riboswitch candidate encountered that was associated with a large and disparate collection of genes. Genetic context of a riboswitch candidate can supply valuable clues regarding ligand identity because the gene located directly downstream from each class representative nearly always is relevant to the natural ligand. Additionally, each organism with an example of a certain riboswitch class must synthesize, import, or otherwise encounter the riboswitch ligand for gene expression to be modulated.

Unfortunately, the diversity of genes associated with GEMM motif RNAs initially confounded efforts to identify ligand candidates for testing because it was unclear how to choose from among the long list of possible ligands relevant to many dozens of different genes. It was soon recognized (Sudarsan et al. 2008) that many associated genes were related to the production, degradation, or signaling networks of cyclic di-guanosine monophosphate (c-di-GMP). Indeed, most members of the GEMM RNA collection appear to be highly selective sensors for c-di-GMP, and control a diversity of genes related to various physiological states of bacteria, including cell motility and biofilm formation (Sudarsan et al. 2008).

It was later discovered that rare variants of what are now called c-di-GMP-I riboswitches have altered their specificity to sense another type of circular nucleotide signaling molecule called c-AMP-GMP (Kellenberger et al. 2015; Nelson et al. 2015). Thus, the original collection of GEMM RNAs represented RNA aptamers for at least two closely related signaling molecules. Although the majority of the original GEMM RNA representatives respond to c-di-GMP, the variant c-AMP-GMP riboswitches regulate different types of genes. Therefore, the comingling of genomic contexts further complicated the formation of initial hypotheses regarding ligand identity. Casually, we use the term “snugglers” to describe the rarer member of a comingled riboswitch collection, because they closely conform to the consensus model for the predominant riboswitch class and carry only subtle nucleotide differences that alter their ligand specificity. We now predict that such riboswitch variations are a widespread phenomenon (e.g., see Weinberg et al. 2017b), and several additional validated examples will be described below.

Although c-di-GMP had been discovered to function as a signaling molecule over three decades earlier (Ross et al. 1987), and some of its regulatory targets had already been identified (Simm et al. 2004; Ryjenkov et al. 2006), the c-di-GMP regulon was not well understood when the GEMM RNA motif was first reported (Weinberg et al. 2007). Thus, uncovering the c-di-GMP-I riboswitch class provided insight into the numerous genes involved c-di-GMP signaling as well as the wide variety of bacterial species that use this signaling molecule. Likewise, identifying the ligands for other riboswitch classes that sense signaling molecules or sense ligands whose biological impacts are only partly understood can immediately provide information on the functions of genes regulated by the riboswitch. Establishing the function of c-di-GMP-I and c-AMP-GMP riboswitches therefore highlights a key motivation for pursuing the functions of orphan riboswitches.

### *crcB* motif RNAs: F^−^ riboswitches

When first reported, the *crcB* motif appeared to be a great candidate for a riboswitch aptamer (Weinberg et al. 2010). Before this, only examples of the TPP riboswitch class had been convincingly observed to occur outside of the bacterial domain of life (Sudarsan et al. 2003b). Also, searches for conserved RNA elements in plants (Hammond et al. 2009) and fungi (Li and Breaker 2017) also failed to reveal additional strong riboswitch candidates, suggesting riboswitches might be rare outside of the eubacterial domain of life. Interestingly, *crcB* motif RNAs are widespread in bacterial and archaeal species (Weinberg et al. 2010). At first, it appeared that the major obstacle to identify the ligand for *crcB* RNAs would again be the wide variety of genes found downstream from this riboswitch candidate. However, the larger hurdle was that the functions of certain genes in the regulatory network of this orphan riboswitch class were misannotated. Very frequently, genome annotations are made based on sequence similarity between the protein encoded by the gene of interest and a protein whose function has been experimentally established. Unfortunately, these predictions can be wrong, but might be taken as fact by researchers. This issue has interfered with efforts to study several other former orphan riboswitch classes, and likely will continue to impede experimental progress for many of the current candidates.

The *crcB* motif was predicted to control the expression of genes encoding K^+^ and Cl^−^ transporters, as well as DNA repair proteins (Weinberg et al. 2010). After the identification of the c-di-GMP riboswitch class, which also regulates a wide variety of processes, it seemed plausible that the *crcB* motif might also be involved in a signaling network mediated by a second messenger. To test the hypothesis that this signaling molecule might be another nucleotide derivative, several synthetic dinucleotides (analogs of their commercially unavailable cyclic dinucleotide isomers) were evaluated for binding by the *crcB* aptamer (Baker et al. 2012) using an in vitro assay called in-line probing (Soukup and Breaker 1999; Regulski and Breaker 2008). Curiously, many of these samples triggered structural changes in the RNA aptamer, but only when the molecules were obtained from one commercial source and not another (Baker et al. 2012). Eventually, it was determined that the dinucleotide compounds themselves had no effect on the RNA. Rather, a contaminant produced during their chemical synthesis, tetra-n-butylammonium fluoride, was responsible for this signal. When fluoride-containing compounds were tested in other forms, such as potassium fluoride and sodium fluoride, it was confirmed that F^−^ was the cognate ligand for the *crcB* orphan riboswitch (Baker et al. 2012).

The serendipitous discovery that fluoride is the ligand for this RNA motif revealed the large “super-regulon” for this toxic ion. The *eriC* gene, which is commonly associated with the *crcB* RNA motif, encodes a protein homologous to known Cl^−^ transporters (Dutzler et al. 2002; Matulef and Maduke 2007). However, the EriC transporters whose expression is regulated by F^−^ riboswitches have differences in the identities of certain amino acids that permit selective F^−^ ion export (Baker et al. 2012; Stockbridge et al. 2012). The misannotation of these EriC transporters as canonical Cl^−^ transporters, as well as a general lack of awareness of the fluoride toxicity mitigation mechanisms used by cells, complicated the initial efforts to experimentally validate *crcB* motif RNAs as riboswitches. The discovery of F^−^ as the ligand for this orphan riboswitch class contributed to our understanding of fluoride resistance mechanisms in a number of organisms (Li et al. 2013; Smith et al. 2015; Stockbridge et al. 2015) and provided evidence that, surprisingly, RNA can selectively bind a small, negatively charged ion (Ren et al. 2012).

### *ydaO* motif RNAs: c-di-AMP riboswitches

The *ydaO* motif RNA was another member of the original orphan riboswitch candidates list (Barrick et al. 2004). This RNA motif exhibited all of the hallmarks of a riboswitch class, and it was again associated with a large and diverse collection of genes. Predicted roles for the protein products of these genes included K^+^ transporters, amino acid transporters, and other proteins involved in cell wall remodeling or osmotic shock response. The major obstacle was that the cognate ligand eventually paired with this riboswitch class had not been discovered to exist in any natural system at the time the RNA motif was discovered.

A multitude of specific hypotheses regarding the function of *ydaO* motif RNAs were pursued over the years (e.g., Watson and Fedor 2012). In addition, unbiased approaches were also used in attempts to identify the mystery ligand, including genetic selection (Block et al. 2010) and testing of cellular extracts as a rich source of small-molecule metabolites and ions (Nelson et al. 2013). A key advance was made when yeast extract was found to contain a compound that consistently caused structural modulation of the RNA when examined by in-line probing. Fractionation of the extract followed by mass spectrometry analysis revealed that the compound responsible for these effects contains an AMP moiety. Although AMP is not directly recognized by members of this RNA class (Block et al. 2010), cyclic di-adenosine monophosphate (c-di-AMP), a signaling molecule first reported in 2008 (Witte et al. 2008), is bound by the RNA with picomolar affinity (Nelson et al. 2013).

The outcomes of this study closely parallel the results of the studies on c-di-GMP-I and c-AMP-GMP riboswitches described above. The validation of the c-di-AMP riboswitch class as a widespread receptor for this signaling molecule helped strengthen the connection between c-di-AMP and osmotic shock responses (Nelson et al. 2013). In addition, the collection of c-di-AMP riboswitches provided clues regarding the functions of a large variety of proteins associated with the c-di-AMP regulon and demonstrated the widespread use of this signaling molecule by many diverse bacterial species.

### *pfl* motif RNAs: ZTP riboswitches

Unlike many other orphan riboswitch candidates, the *pfl* RNA motif had gene associations that were relevant to well-established metabolic pathways (Weinberg et al. 2010; Meyer et al. 2011). The genes most commonly associated with this riboswitch candidate were known to encode enzymes at the intersection of de novo purine biosynthesis and the production of 10-formyl-tetrahydrofolate (10f-THF). Positioned at this metabolic intersection is the molecule 5-aminoimidazole-4-carboxamide ribonucleotide (AICAR, also known as ZMP) (Bochner and Ames 1982). Immediately after the *pfl* motif was first identified, ZMP was examined by in-line probing to evaluate whether it could be directly recognized by a representative *pfl* RNA (Weinberg et al. 2010). These initial experiments, along with the examination of additional ligand candidates (Meyer et al. 2011), did not yield any evidence of ligand-mediated RNA structural changes. Thus, the *pfl* RNA motif was considered an orphan riboswitch candidate for several years.

Unfortunately, these early experiments used a *pfl* motif RNA representative that was partly misfolded in vitro and failed to adopt the correct aptamer structure necessary for ligand recognition. This technical issue was eventually recognized and was resolved by redesigning the RNA construct. Once a properly folding RNA was identified, biochemical and genetic evidence for riboswitch function triggered by ZMP and its triphosphate derivative ZTP was quickly collected (Kim et al. 2015). The experimental validation of the ZTP riboswitch class, and its association with numerous folate and purine metabolic genes, also confirmed a disputed hypothesis from decades earlier that ZTP functions as an “alarmone” to signal 10f-THF deficiency (Bochner and Ames 1982; Rohlman and Matthews 1990). This uncertainty was thus dispelled by the discovery of riboswitches that sense this unusual bacterial signaling molecule.

### *yybP* motif RNAs: Mn^2+^ riboswitches

The *yybP* RNA motif is one of several well-conserved, widespread, and extremely abundant RNA elements that spent many years classified as an orphan riboswitch candidate (Barrick et al. 2004; Meyer et al. 2011). This motif is found upstream of various genes encoding putative cation transporters that are ambiguously annotated. Similar to the circumstances encountered when pursuing the experimental validation of F^−^ riboswitches (Baker et al. 2012), the limiting factor in identifying the ligand for members of this orphan riboswitch class was the undetermined specificity of transporter proteins encoded by its associated genes.

A key advance was made when one of the genes predicted to be regulated by this RNA motif in *E. coli* was determined to code for a Mn^2+^ exporter (Waters et al. 2011). Support for riboswitch function was derived from various genetic, biochemical and biophysical experiments, which confirmed direct and selective interactions between certain *yybP* orphan riboswitch RNAs and Mn^2+^ (Dambach et al. 2015; Price et al. 2015). These Mn^2+^-sensing aptamers showcase the ability of RNA to exhibit high specificity for a particular ion over other ions of similar size and charge, some of which are at higher concentrations in the cell.

The validation of Mn^2+^ as the ligand for this riboswitch class also suggests that genes encoding other putative transporters in its regulatory network may also be specific for Mn^2+^. However, it also seems likely that there are some common *yybP* motif RNA variants currently grouped with Mn^2+^ riboswitches that have altered their ligand specificity and respond to other divalent metal ions of biological importance (KR Perkins, RR Breaker, in prep.). The existence of aptamers that have altered their binding pockets to sense different divalent metal ions would help explain some of the diverse genes associated with *yybP* motif RNAs that are not consistent with Mn^2+^ sensing. Furthermore, the collection of *yybP* motif RNAs constitutes one of the most abundant bacterial riboswitches. Perhaps its widespread distribution reflects the utility of this architecture for selectively sensing Mn^2+^ or sensing other divalent metal ions by tuning its binding site through natural mutation.

### *yjdF* motif RNAs: azaaromatic riboswitches

The *yjdF* motif is almost exclusively found upstream of the gene of the same name (Weinberg et al. 2010), but unfortunately there is no established function for the YjdF protein. Thus, two different experimental approaches were used to investigate the potential function of this RNA motif. For the first approach, extremely rare gene associations were considered, as they hinted that the ligand might have some connection to nicotinamide adenine dinucleotide (NAD^+^) or FMN metabolism. These coenzymes and their derivatives were examined for in vitro binding to *yjdF* RNAs, which unexpectedly revealed that diverse flavin analogs are bound by *yjdF* RNAs with relatively high affinity (Li et al. 2016). Mutations to highly conserved nucleotides disrupt associations with these ligands, indicating that this promiscuous binding was unlikely to be due to nonspecific interactions.

For the second approach, various growth media conditions supplemented with various chemical additives were used in an attempt to find cellular conditions that activate *lacZ* reporter gene fused to a *yjdF* riboswitch. Specifically, a *B. subtilis* riboswitch-reporter strain was grown in ∼2000 conditions sampled by a growth media library. Only five supplemented compounds triggered the *yjdF* RNA-*lacZ* reporter, all of which were large, planar, nitrogenous, polycyclic molecules (Li et al. 2016). The hits from the screen, as well as the flavin derivatives identified through additional binding assays, share little resemblance with one another except that they all fall into the general category of “azaaromatic” molecules. Although these results do not reveal a single ligand with clear biological relevance, they do demonstrate that *yjdF* RNAs function as genetic “ON” riboswitches with unusually broad specificity.

Most known riboswitch classes demonstrate high selectivity for their cognate ligand over other similar molecules. The *yjdF* riboswitch class might have evolved to function as an RNA-based sensor with an intentionally broad ligand specificity. Broad substrate specificities previously have been observed for certain transporter proteins, yet this phenomenon has not been observed for any previous riboswitch class. Intriguingly, in the instances where the *yjdF* gene is not associated with the riboswitch, a gene for PadR regulatory proteins, known for their broad ligand specificity (De Silva et al. 2005), is present. Thus, azaaromatic riboswitches might be functionally similar to regulatory proteins also known to broadly recognize planar, hydrophobic molecules.

If true, then the *yjdF* riboswitch class could respond to a diverse class of natural compounds that might be toxic to bacteria at high concentrations and increase expression levels of an exporter protein (YjdF) to eject these toxins. However, the possibility remains that one specific, biologically relevant compound is the true ligand for *yjdF* motif RNAs. For this reason, the azaaromatic riboswitch class still has lingering, quasi-orphan status, which might only be resolved through experimental demonstration of the function of the YjdF protein or through high-resolution structural analysis of *yjdF* RNAs bound to various azaaromatic compounds.

### *ykkC* motif RNAs: guanidine riboswitches, and much more

The *ykkC* RNA motif was the last of the original set of orphan riboswitches (Barrick et al. 2004) to be experimentally associated with a ligand (Nelson et al. 2017). The eclectic mixture of genes predicted to be regulated by the cognate ligand for this orphan riboswitch candidate included those encoding urea carboxylases, multidrug efflux pumps, nitrate/sulfate/bicarbonate transporters, de novo purine biosynthesis enzymes, branched-chain amino acid biosynthesis enzymes, and many more (Barrick et al. 2004; Meyer et al. 2011). Two additional RNA motifs were later discovered that are both found upstream of a subset of these same, seemingly unrelated, genes. While neither of these additional riboswitch candidates, termed mini-*ykkC* (Weinberg et al. 2007) and *ykkC*-III (Weinberg et al. 2010), shares sequence or structural homology with one another or to the original *ykkC*-I motif, the extensive overlap in the genes associated with all three *ykkC* RNA motifs indicated that they would likely perform analogous functions by sensing the same cognate ligand. Numerous problems conspired to hinder validation of the *ykkC* family of orphan riboswitch candidates, including the wide variety of genes controlled, gene misannotations, and the abundant presence of variants with substantial differences in their cognate ligand identity.

Over the course of a decade, dozens of ligands were tested in vitro for binding to *ykkC* motif RNAs, and in vivo genetic screens were performed to attempt to find conditions that would activate a *ykkC* RNA-*lacZ* reporter fusion construct—all without any success. Eventually, a *B. subtilis* strain carrying a *ykkC* reporter construct was subjected to the same growth media screen described above for the azaaromatic riboswitch class (Li et al. 2016), which revealed one specific hit: guanidine (or more likely its cationic form, guanidinium) (Nelson et al. 2017). This result was initially puzzling because guanidine is a chaotropic agent commonly used to denature proteins in a laboratory setting. However, follow-up experiments confirmed that the *ykkC* reporter construct responds to guanidine in a dose-dependent manner. Furthermore, guanidine is bound by *ykkC* RNAs in vitro (Battaglia et al. 2017; Nelson et al. 2017; Reiss et al. 2017), whereas similar molecules including urea and arginine are rejected.

Representatives of both the mini-*ykkC* and *ykkC*-III RNA motifs also specifically bind guanidine in vitro (Sherlock and Breaker 2017; Sherlock et al. 2017). The mini-*ykkC* motif primarily consists of two identical stem loops and, for this reason, was initially only considered a weak orphan riboswitch candidate (Weinberg et al. 2010). Despite their simplicity, mini-*ykkC* RNAs, now classified as guanidine-II riboswitches, bind two guanidine molecules cooperatively (Huang et al. 2017a; Reiss and Strobel 2017; Sherlock et al. 2017). The guanidine-II riboswitch class is one of the simplest with regard to its size and complexity. Using yet another distinct architecture, *ykkC*-III motif RNAs bind a single guanidine molecule and have been renamed the guanidine-III riboswitch class (Huang et al. 2017b; Sherlock and Breaker 2017).

Ample experimental evidence supports the conclusion that many guanidine riboswitches exist, but there were two puzzling issues to consider further. First, guanidine had not previously been implicated as a biologically relevant metabolite, and its natural sources still remain obscure. However, guanidine is toxic to *B. subtilis* cells at high concentrations (Nelson et al. 2017), and the small multidrug resistance (SMR) transporters associated with guanidine riboswitches appear to expel guanidine to reduce its toxicity (Kermani et al. 2018; Higgins et al. 2019). Several other types of genes under the regulatory control of *ykkC* RNAs express proteins that perform functions related to guanidine detoxification. Specifically, enzymes annotated as urea carboxylases whose expression is controlled by guanidine riboswitches demonstrate greater catalytic efficiency with guanidine as a substrate compared to urea (Nelson et al. 2017). Therefore, the true purpose of these carboxylases and their associated protein partners is probably to degrade guanidine into ammonia and carbon dioxide.

A second puzzling issue relates to the observation that many types of genes associated with *ykkC* RNAs, including those encoding proteins related to de novo purine and branched-chain amino acid (BCAA) biosynthesis, are extremely well understood and have no apparent link to guanidine detoxification. Experimental analyses determined that *ykkC* RNAs found upstream of purine or BCAA biosynthesis genes do not bind guanidine (Nelson et al. 2017). Whereas the majority of *ykkC* motif RNAs (subtype 1) constitute the guanidine-I riboswitch class, the remaining *ykkC* motif RNAs (subtype 2) have nucleotide changes in the binding pocket that alter the ligand specificity and represent several distinct riboswitch classes. This RNA collection, which includes the largest group of variants found to date, carry out a surprising diversity of molecular recognition and gene control functions as described below.

### *ykkC* subtype 2 motif RNAs: riboswitches for ppGpp, PRPP, (d)NDPs, and one orphan

The genes associated with *ykkC* subtype 2 RNAs primarily code for enzymes involved in amino acid and de novo purine biosynthesis, enzymes of the nucleoside diphosphate linked to X (NUDIX) or haloacid dehalogenase-like (HAD) hydrolase families, and transporters annotated as EamA or TauE. Again, this disparate set of gene functions initially hindered ligand identification for the remaining representatives of the *ykkC* orphan riboswitch collection. Surprisingly, further bioinformatic and experimental data revealed that *ykkC* subtype 2 consists of at least four distinct riboswitch classes (Sherlock et al. 2018a). Each of these riboswitch candidate classes, termed subtypes 2a through 2d, has distinct differences in the nucleotide positions corresponding to the binding pocket of guanidine-I aptamers that were predicted to alter ligand specificity. At this time, the ligands for subtypes 2a (Sherlock et al. 2018a), 2b (Sherlock et al. 2018b), and 2c (Sherlock et al. 2019) have been established as noted below. The ligand for 2d has yet to be experimentally determined.

Subtype 2a *ykkC* RNAs regulate the expression of genes for amino acid biosynthesis in response to guanosine tetraphosphate (Sherlock et al. 2018a), which is commonly known as ppGpp or “magic spot.” This nucleotide signaling molecule is nearly ubiquitous in bacteria and mediates amino acid starvation and cell envelope stresses, among other metabolic adaptations (Potrykus and Cashel 2008; Geiger et al. 2014; Hauryliuk et al. 2015). When certain amino acid levels drop below a critical concentration in the cell, uncharged tRNAs accumulate and the ppGpp synthase RelA is activated (Wolz et al. 2010). To overcome amino acid depletion, the ppGpp riboswitch class activates the expression of genes whose protein products promote the biosynthesis of valine, leucine, and isoleucine (branched chain amino acids), as well as glutamate.

Subtype 2b *ykkC* RNAs regulate the expression of de novo purine biosynthesis genes in response to phosphoribosyl pyrophosphate (PRPP), a molecule necessary for the synthesis of all nucleotide monomers (Sherlock et al. 2018b). High levels of PRPP, the activated ribose precursor for purine biosynthesis, trigger this riboswitch class to turn on all of the genes necessary to produce inosine monophosphate, which is at the branch point of AMP and GMP biosynthesis. Frequently, a PRPP aptamer is located directly adjacent to a guanine aptamer (Mandal et al. 2003), and the two aptamers share a single expression platform (terminator stem) to create a complex tandem riboswitch system that regulates purine metabolism according to the relative abundance of both the guanine and PRPP ligands (Sherlock et al. 2018b).

Subtype 2c *ykkC* RNAs regulate the expression of genes encoding NUDIX and HAD-like hydrolases in response to nucleoside diphosphates (Sherlock et al. 2019). Representatives of this riboswitch class selectively recognize adenosine and cytidine diphosphate in both the ribose and deoxyribose forms. The NUDIX and HAD-like hydrolases whose expressions are predicted to increase in the presence of these nucleotide ligands are known to cleave the phosphodiester bonds of a variety of nucleotide substrates (Lu et al. 2005; McLennan 2006). The precise identity of the ligand(s) for members of this riboswitch class remains somewhat unclear due to the ability of representatives to recognize multiple RNA and DNA nucleoside-5′-diphosphate molecules with similar affinities. We speculate that members of the *ykkC* subtype 2c class might respond more broadly to the nucleoside diphosphate pool. If true, ligand binding would activate the expression of enzymes that maintain the balance of phosphorylated nucleotide concentrations.

Subtype 2d *ykkC* RNAs regulate the expression of genes encoding transporters annotated as EamA (originally called phn_DUF6) and TauE (also known as SafE) (Nelson et al. 2017; Sherlock et al. 2019) in response to a ligand that remains undetermined at this time. Thus, at least one more orphan riboswitch class is present among the original *ykkC* motif RNAs. A variety of problems had to be overcome to experimentally establish the four validated classes from the larger *ykkC* motif collection. Particularly important was the realization that the *ykkC* RNA motif represents multiple riboswitch classes that sense fundamentally different ligand molecules, which was surprising and unprecedented. Of course, variant aptamers for other riboswitch classes had previously been uncovered, but the structures of the ligands for those variant class had always been highly similar to that of the predominant classes such as guanine (adenine and 2′-dG) (Mandal and Breaker 2004; Kim et al. 2007; Weinberg et al. 2017b), MoCo (WCo) (Regulski et al. 2008), and c-di-GMP (c-AMP-GMP) (Sudarsan et al. 2008; Kellenberger et al. 2015; Nelson et al. 2015).

The identification of subtype 2 *ykkC* RNAs and the validation of the ligands for several of these variant riboswitch classes have provided a new perspective into the evolution of an RNA scaffold that is likely very ancient in origin. A riboswitch class that responds to ppGpp, a common nucleotide signaling molecule that had long been postulated as a riboswitch ligand (Breaker 2010; Kim et al. 2015; Nelson and Breaker 2017), has now been identified. A riboswitch class for PRPP, another fundamental RNA biosynthetic precursor, was found to regulate RNA monomer biosynthesis, which has strong RNA World implications. Subtype 2c RNAs selectively recognize 5′-diphosphorylated nucleotides, even though they are less selective for the sugar and nucleobase types.

Together, these riboswitch classes are immensely intriguing because their consensus models are nearly identical, and yet each excludes the binding of the other's ligands (e.g., see Knappenberger et al. 2018; Peselis and Serganov, 2018). To allow for recognition of chemically diverse compounds by the *ykkC* collection of riboswitches, the nucleotide changes that enable the formation of the unique binding pockets between different subtypes are drastic, although they were initially undetected among the many other highly conserved nucleotides (Barrick et al. 2004). Thus, the sequences and structural features common to all *ykkC* motif RNAs appear to have been exploited to create multiple RNAs comprising the same overall architecture, but that have the ability to recognize a surprising diversity of small molecules. It is reasonable to expect that establishing the ligand specificity for subtype 2d *ykkC* RNAs eventually will reveal even more new and exciting biology.

### *thiS* motif RNAs: riboswitches forHMP-PP

**FIGURE 4.**
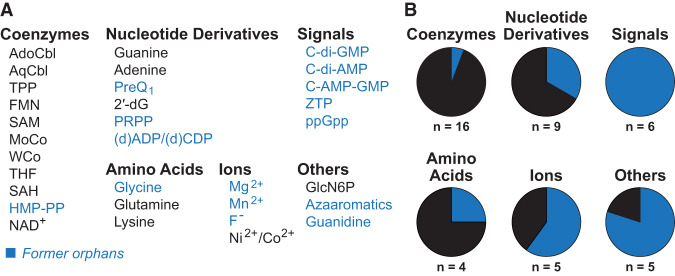
The unequal distribution of ligands sensed by former orphan riboswitch candidates. (*A*) Ligands of all experimentally validated riboswitch classes. Ligands in blue designate those sensed by at least one former orphan riboswitch class. (*B*) The proportion of validated riboswitch classes that were once considered orphan riboswitch candidates (blue). Charts are organized by the type of ligand sensed, where *n* is the number of classes in the group indicated. Note that some ligands (SAM, THF, preQ_1_, 2′-dG, Mg^2+^, glutamine and guanidine) are sensed by multiple distinct riboswitch classes. This accounts for the difference between the total number of riboswitch classes and the number of ligands sensed by riboswitches as reported in panel *A*.

The pathways for the biosynthesis of the common enzyme cofactors have been extensively documented, and candidate riboswitch classes associated with these pathways are almost always quickly validated experimentally, without spending time on the orphans list (Fig. 4). One exception was a riboswitch candidate originally called *thiS* (Stav et al. 2019). Representatives of this class are always associated with genes coding for enzymes that synthesize the TPP biosynthetic intermediate hydroxyethyl-thiazole phosphate (HET-P) (Begley et al. 1999). In some instances, *thiS* motifRNAs are found in tandem arrangements with TPP riboswitches, suggesting that the ligand must somehow relate to this ubiquitous coenzyme.

Despite these clues, ligand binding assays initially failed to reveal the natural ligand for this riboswitch class. However, the use of riboswitch reporter-fusion constructs and bacterial strains with knockouts of genes in the TPP biosynthetic pathway revealed that another intermediate, HMP-PP, was the ligand (Atilho et al. 2019; Stav et al. 2019). The riboswitch senses the buildup of HMP-PP to activate the expression of genes involved in producing HET-P, which together form the final coenzyme product.

The biochemical assays were hindered by the fact that this riboswitch has a unique architecture wherein the aptamer is almost entirely embedded within the intrinsic terminator stem that serves as the expression platform (Atilho et al. 2019; Stav et al. 2019). This arrangement means that when RNA constructs including the entire terminator stem are tested, the strength of this stem causes the terminator to dominate and preclude the formation of the structure needed to bind HMP-PP. In the natural setting for the riboswitch, RNA polymerase makes a progressively longer RNA transcript, and HMP-PP binds at precisely the right time when the transcript can form the aptamer structure, but before the terminator stem can dominate. To resolve this architectural problem, mutant constructs of just the right length had to be created to observe HMP-PP binding in vitro (Atilho et al. 2019).

## OBSTACLES TO FUTURE ORPHAN RIBOSWITCH VALIDATION

**FIGURE 5.**
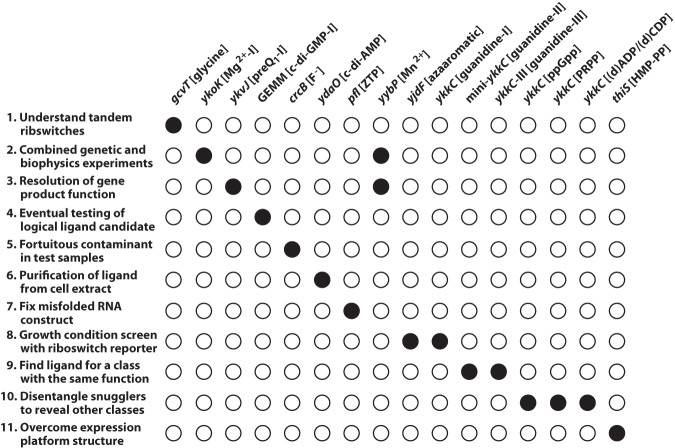
Special efforts needed to identify the ligands for orphan riboswitch classes. Former orphan riboswitch classes are listed at the *top* in chronological order from *left* to *right*, relative to their experimental validation. Filled circles indicate which unusual strategy or occurrence was needed to advance the validation project. Note that *yybP* motif RNAs (Mn^2+^ riboswitches) involved two categories for experimental validation to progress successfully.

Presented with all of the orphan riboswitch examples that have since been experimentally validated, we can review all the major factors that hindered ligand identification and the approaches used to overcome them. These validation roadblocks fall into numerous categories (Fig. 5) and include the key challenges discussed in detail below. There are currently dozens of orphan riboswitch candidates remaining to be analyzed (Corbino et al. 2005; Weinberg et al. 2007, 2010, 2017a,b; Greenlee et al. 2018), and therefore new strategies to overcome these limitations would help drive forward riboswitch validation research.

### Bioinformatic analysis of a riboswitch aptamer

Perhaps the greatest barrier to the future of riboswitch ligand discovery is that not every orphan riboswitch candidate is assured to be a metabolite- or inorganic ion-binding riboswitch class. Some of these RNA motifs might represent other types of *cis*-regulatory elements that respond to ligands made of RNA or protein, or that respond to changes in temperature such as RNA thermometers (Kortmann and Narberhaus 2012). The functions of these other types of regulatory RNA systems are not always best elucidated by using the in vitro binding assays typically used for riboswitch ligand validation. If this element is a binding site for a protein or RNA genetic factor, then biochemical assays seeking evidence for direct metabolite binding will fail to yield the desired evidence. The candidate might then remain on the orphan riboswitch list when it is not functioning as a typical metabolite- or ion-binding riboswitch.

Using bioinformatic analyses to seek evidence of riboswitch function can be further complicated by rare examples of trans-acting riboswitch representatives, which sometimes regulate an adjacent gene in the conventional manner and then diffuse to interact with distal mRNAs (Loh et al, 2009) or proteins (DebRoy et al. 2014; Mellin et al. 2014). Such RNAs could carry a ligand-binding aptamer that is distinct from a region that either base-pairs to its target mRNA or serves as a protein binding site. In these instances, sequence and structure conservation could be due to functions other than binding a small molecule or metal ion ligand.

Another challenge associated with the bioinformatic analysis of riboswitch candidates is obtaining an accurate consensus sequence and structural model for the aptamer domain. For example, the initial consensus models for c-di-GMP (Weinberg et al. 2007), SAM-VI (Mirihana Arachchilage et al. 2018), and *ykkC* subtype 2 riboswitches (Nelson et al. 2017) were missing important structural features. In these cases, either the RNA constructs tested by in-line probing were fortuitously long enough to encompass the entire aptamer (Sudarsan et al. 2008; Mirihana Arachchilage et al. 2018), or additional conservation was found by comparative sequence analysis at a later time and the RNA constructs used for evaluation were adjusted accordingly (Sherlock et al. 2018a,b, 2019).

Defining the exact start and end points for an RNA motif can be problematic when relying solely on bioinformatic data, especially for riboswitch candidates with few representatives that exhibit modest sequence diversity. Members of the current list of orphan riboswitches might have already been subjected to binding assays with the cognate natural ligand, but binding was not observed because a portion of the aptamer was missing. This notion was supported by the recent effort to update the bioinformatic data for certain orphan riboswitch candidates, which revealed additional conserved sequence and structures for the *speF* and *serC* RNA motifs (Greenlee et al. 2018).

### Inaccurate or missing annotations for riboswitch-associated genes

In most published riboswitch validation studies, determining the cognate ligand for a riboswitch class relies almost entirely on analyzing the identity of the genes in its regulatory network. Missing information regarding the function of the protein products from these genes frequently causes a motif to spend more time on the orphan riboswitch candidate list. In some cases, the function of the proteins whose expression is controlled by the riboswitch class is undetermined. In other cases, the predicted function of the genes might only be generally categorized, or the gene annotation could be incorrect and therefore misleading.

Aside from some biosynthesis pathways and certain other regulatory systems that are particularly well characterized, it is extremely difficult to determine which annotations for protein functions and substrates are accurate, especially for transporters and broad enzyme families. Although this increases the difficulty for researchers seeking to validate the ligand for a given riboswitch class, successful validation efforts can yield interesting knowledge about the proteins whose genes are under riboswitch regulation.

### Perplexing gene associations

Some current orphan riboswitch candidates are associated with a vast breadth of genes involved in a variety of processes that may not initially appear to be related to one another. Some of these riboswitch candidates could respond to signaling molecules that have yet to be discovered, especially because many orphan riboswitch classes are narrowly distributed phylogenetically. There might be little or no information available about the pathways and signaling systems specific to the organisms harboring these riboswitch candidates.

Strikingly, every riboswitch class that senses a signaling molecule was formerly classified as an orphan riboswitch or was uncovered from the collection of riboswitch representatives originally assigned to another class. In contrast, only two of the numerous riboswitch classes that sense coenzymes and amino acids were ever considered orphan riboswitch candidates (Fig. 4). These latter riboswitches, for glycine and HMP-PP, only resisted initial attempts at biochemical validation due to their unusual architectures (Mandal et al. 2004; Atilho et al. 2019; Stav et al. 2019). In general, amino acid and coenzyme biosynthesis pathways have been extensively studied, whereas certain signaling networks and metal ion homeostasis processes represent complex topics of study that are still being explored. The processes regulated by many former and likely also current orphan riboswitch classes parallel our collective lack of knowledge in certain areas of bacterial physiology. Therefore, we believe that experimental pursuit of these RNAs can yield valuable information regarding previously undetermined gene networks and pathways.

### Variant riboswitch aptamers

As noted above, the collection of RNAs encompassed by the *ykkC* motif (Barrick et al. 2004) presented the most extreme case encountered to date wherein riboswitch variants are intermixed within what originally appeared to be a single riboswitch candidate class (Nelson et al. 2017; Sherlock et al. 2018a,b, 2019). The diverse set of genes associated with *ykkC* motif RNAs made it particularly difficult to come up with a single best hypothesis for the ligand identity. The fact that some genes code for proteins with no apparent functional relationship to others in the collection should have served as early evidence for the existence of more than one riboswitch class. Although both ppGpp and PRPP had been identified as likely ligand candidates, binding assays did not give positive results because all experiments made use of a *ykkC* motif representative that we now know is selective for guanidine. If the *ykkC* motif is unique regarding its broad range of aptamers and their corresponding ligands, then the difficulties experienced during validation studies will not be a problem for future riboswitch validation projects. However, many orphan riboswitch candidates are upstream of genes that code for proteins with diverse functions, which might indicate the existence of multiple riboswitch classes with distinct ligand specificities. To overcome the binding assay problem encountered with *ykkC* motif RNAs, it is advisable to test ligand candidates for binding using RNA representatives that are naturally found upstream of a specific gene that led to the ligand hypothesis.

Although the abundance of variants among the *ykkC* motif population caused considerable experimental problems, the benefit from persisting with the validation efforts are also large. The first biological receptors specific for guanidine were identified, and these findings indicate the widespread presence of guanidine in bacteria when this compound was not previously considered to be biologically relevant. The source of guanidine in cells, the characterization of pathways to diminish its toxic effects, as well as other mysteries surrounding guanidine biology await further investigation. Four additional riboswitch classes also have been uncovered, and the functions of several protein families have now been properly assigned. Perhaps similar opportunities are associated with the list of the orphan riboswitch classes whose ligands remain to be identified.

### Technical challenges of experimental riboswitch analysis

As mentioned above, determining the exact length of a riboswitch aptamer can be a bioinformatic challenge that also impedes experimental analysis. Although it might be prudent to test the entire noncoding region harboring a riboswitch candidate, this strategy can be taken too far. The inclusion of nucleotides beyond the aptamer domain could permit the formation of competing structures that are naturally formed by the adjoining expression platform (e.g., Wickiser et al. 2005). Even when a riboswitch aptamer is clearly defined, a particular representative construct of the RNA motif might still misfold, leading to false negative results from in vitro binding experiments. Although no single foolproof strategy exists for in vitro examination of orphan riboswitch candidates, the analysis of multiple representative constructs with varying amounts of additional flanking sequence might help overcome these problems.

In addition to the difficulties associated with testing RNAs in vitro, certain ligand molecules and elemental ions can present technical challenges. Many charged small molecules and ions can induce nonspecific changes to RNA structures, which must be accounted for when evaluating these ligand candidates. Misleading evidence for ligand binding by RNAs likely has been collected for positively charged compounds such as aminoglycoside antibiotics (Jia et al. 2013) and arginine (Borsuk et al. 2007), which can readily alter the structures of polyanionic RNAs. Thus, care must be taken to avoid false conclusions regarding riboswitch functions (Roth and Breaker 2013; Greenlee et al. 2018). Also, certain metabolites and biochemical intermediates are not readily available or have extremely short half-lives, presenting another type of technical challenge. Overall, a mixture of various in vivo and in vitro approaches can and have been successfully implemented to overcome technical challenges and solve orphan riboswitch candidates.

## METHODS FOR RIBOSWITCH LIGAND DISCOVERY

There are a variety of lessons to be learned from the process of identifying ligands for former orphan riboswitches that might apply to the future validation efforts for other orphan riboswitch candidates (Fig. 5). When a riboswitch candidate motif is found upstream of genes involved in a characterized pathway, the first step is typically to directly test each molecule involved in that pathway for binding using an in vitro binding assay such as in-line probing. Some riboswitches have functions that parallel known protein-mediated regulatory systems, which can provide useful hints regarding ligand identity. Even when the ligand seems obvious based on existing clues, validation experiments can be frustrated by misannotated genes or defective RNA construct designs.

If the gene context provides no useful clues, unbiased approaches might fortuitously reveal the ligand identity and the regulatory role of these riboswitch candidates. The efforts to discover the ligands for *ykkC* (Nelson et al. 2017) and *yjdF* (Li et al. 2016) riboswitch candidates benefited from unbiased screening approaches using in vivo reporter systems, but these are some of the only riboswitch classes out of the more than 45 validated classes for which this approach could have worked. First, the correct ligand or its close analog has to be present in the library screened for there to be a chance that it will be discovered using this method. Furthermore, the ligand must be able to enter the cells, either by diffusion across the membrane, or by active movement through a channel or transporter. Creating a comprehensive library of all possible metabolites, ions, and toxins would take a tremendous amount of time and effort compared to some more feasible, albeit lower throughput, alternatives.

In the cases where the ligand is an internally produced signaling molecule or metabolite, an in vivo reporter gene might only yield positive results when expressed in the native organism. Many of the original orphan riboswitch candidates have natural representatives in either *E. coli* or *B. subtilis*, whereas many promising current orphan riboswitch candidates are not present in these bacterial model organisms. Similarly, as rarer classes of riboswitch candidates are uncovered, it becomes increasingly likely that the ligand will be unique to the few species that carry the RNA motif. In these instances, the ligand might be a compound that has never before been isolated or made by researchers.

Future research efforts toward riboswitch ligand validation might best rely on methods such as testing libraries of compounds, performing phenotypic screens, using cellular extracts or complex mixtures of natural compounds in binding assays, screening for genetic mutants that activate reporter gene expression, or developing approaches to perform high-throughput chromatographic or electrophoretic ligand screening. Regardless, it seems certain that orphan riboswitch classes will be encountered that defy all these powerful experimental approaches.

## CONCLUSIONS

Unfortunately, there is no universal strategy for matching orphan riboswitch candidates with their ligands because each class presents unique challenges. Some obstacles are easy to immediately identify, such as when the riboswitch is associated only with genes of unknown function, or the ligand candidate is not readily obtainable. Other complications can only be realized in retrospect, such as using RNA constructs missing portions of the aptamer or encountering misleading information due to gene misannotation. Sometimes simple luck is needed to achieve an experimental breakthrough. Overall, the limiting factors frequently involve a fundamental lack of knowledge in certain areas of biology, rather than any technical limitations in the experimental methodology used to establish riboswitch function.

Riboswitch classes that were immediately validated upon their bioinformatic discovery invariably regulate biological processes that had been characterized many years or even decades earlier. Although these riboswitch classes are certainly worth investigating, they rarely teach us anything new about the genes they regulate. Conversely, finding the ligands for orphan riboswitch classes has often shed light on unknown or underappreciated aspects of microbiology. When an unbiased strategy leads to the identification of the target for an orphan riboswitch, a link is immediately established between that ligand and every gene whose expression it regulates in each organism in which it is found. Despite the frustrating nature of experimentally pursuing difficult riboswitch candidates, the rewards in terms of the scientific knowledge gained by establishing their functions are worth the efforts. Elucidating the functions of these mysterious RNAs has and will continue to reveal unexplored areas of biology.
